# Supplementary material for: NO Synthesis but Not Apoptosis, Mitosis or Inflammation Can Explain Correlations between Flow Directionality and Paracellular Permeability of Cultured Endothelium
Source: Int J Mol Sci. 2022 Jul 22;23(15):8076. doi: 10.3390/ijms23158076 (PMC9332325; doi:10.3390/ijms23158076)
Supplement: Supplementary file 1 [file ijms-23-08076-s001.zip › ijms-1734204-supplementary.pdf]

**Supplementary Figures**

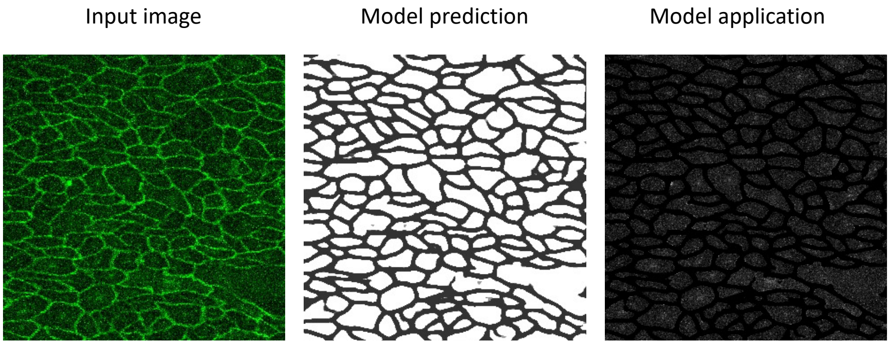


Supplementary Figure S1. Segmentation of anti-VE-cadherin staining of cell borders and its use for quantification of intracellular NF-κB p65. Images show, from left to right, the original VE-cadherin immunostaining, the model prediction of cell borders, and use of the borders to quantify NF-κB p65 immunostaining within the cell.

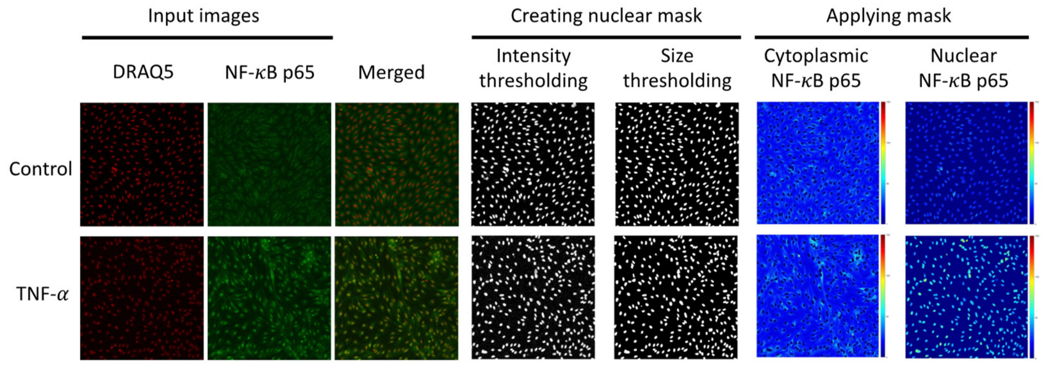


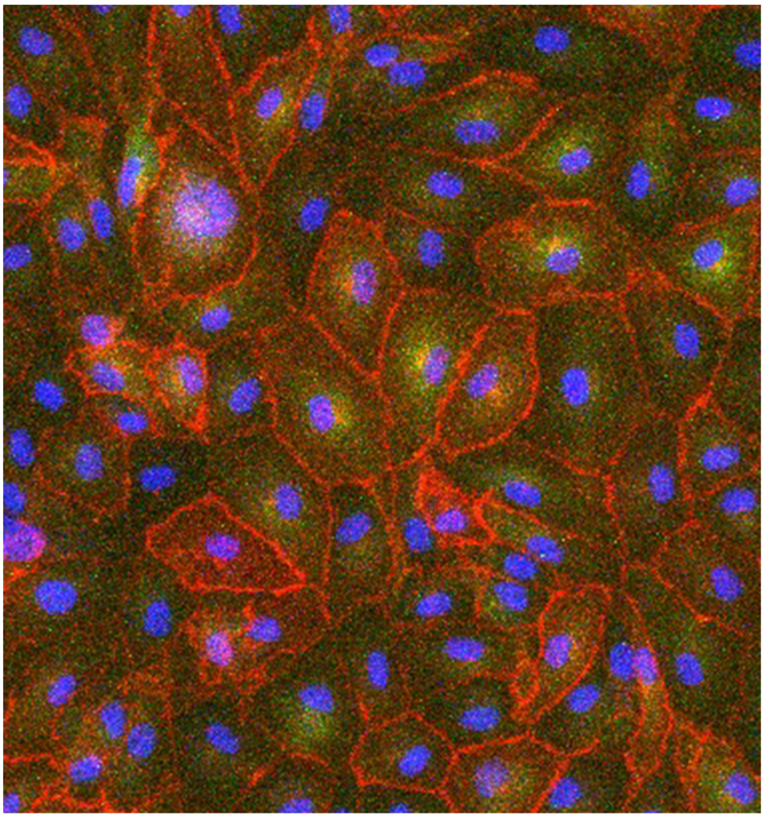

Supplementary Figure S2. (Top) Outline of the methods for quantifying NF-κB p65 translocation. Representative data are shown for cells under control conditions and for cells exposed to TNF-α; more nuclear NF-κB and elongation of the cells are visible in the TNF-α case. (Bottom) A merged image of staining for NF-κB p65 (green), nuclei (blue) and VE-cadherin (red) at higher magnification.

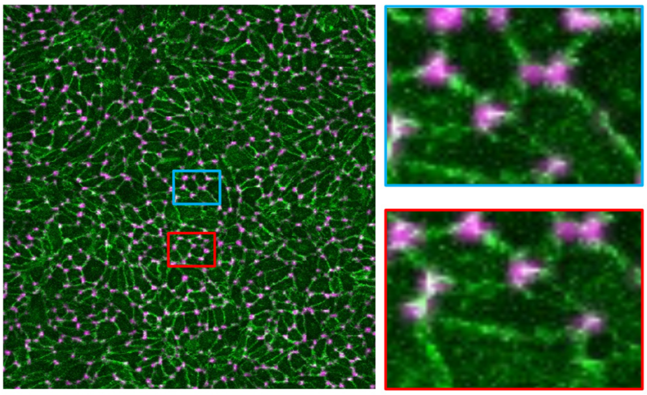

Supplementary Figure S3. Semantic segmentation of tricellular junctions: image of tricellular junctions predicted by the model output (purple) overlaid on immunostaining of VE-cadherin, showing PAEC borders (green), at low magnification (left), and at high magnification for the areas delineated by blue and red boxes (right top and bottom).
